# Glycine Transporter 1 Inhibitors Minimize the Analgesic Tolerance to Morphine

**DOI:** 10.3390/ijms252011136

**Published:** 2024-10-17

**Authors:** Anna Rita Galambos, Nariman Essmat, Péter P. Lakatos, Edina Szücs, Imre Boldizsár, Sarah Kadhim Abbood, Dávid Á. Karádi, Judit Mária Kirchlechner-Farkas, Kornél Király, Sándor Benyhe, Pál Riba, Tamás Tábi, Laszlo G. Harsing, Ferenc Zádor, Mahmoud Al-Khrasani

**Affiliations:** 1 Department of Pharmacology and Pharmacotherapy, Faculty of Medicine, Semmelweis University, Nagyvárad tér 4, H-1085 Budapest, Hungary; galambos.anna@phd.semmelweis.hu (A.R.G.); nariman.gomaa@phd.semmelweis.hu (N.E.); boldizsar.imre2@semmelweis.hu (I.B.J.); abbood.sarah@phd.semmelweis.hu (S.K.A.); karadi.david.arpad@semmelweis.hu (D.Á.K.); kirchlechner.farkas.jmi@gmail.com (J.M.K.-F.); kiraly.kornel@semmelweis.hu (K.K.); riba.pal@semmelweis.hu (P.R.); harsing.laszlo@semmelweis.hu (L.G.H.J.); 2 Center for Pharmacology and Drug Research & Development, Semmelweis University, Üllői út 26., H-1085 Budapest, Hungary; lakatos.peter.pal@semmelweis.hu (P.P.L.); tabi.tamas@semmelweis.hu (T.T.); 3 Department of Pharmacodynamics, Semmelweis University, Nagyvárad tér 4, H-1089 Budapest, Hungary; 4 Institute of Genetics, HUN-REN Biological Research Centre, Temesvári krt. 62, H-6726 Szeged, Hungary; szucs.edina@brc.hu; 5 HUN-REN Biological Research Centre, Institute of Biochemistry, Temesvári krt. 62, H-6726 Szeged, Hungary; benyhe.sandor@brc.hu

**Keywords:** opioid analgesic tolerance, glycine transporter-1, NMDARs, NFPS

## Abstract

Opioid analgesic tolerance (OAT), among other central side effects, limits opioids’ indispensable clinical use for managing chronic pain. Therefore, there is an existing unmet medical need to prevent OAT. Extrasynaptic N-methyl D-aspartate receptors (NMDARs) containing GluN2B subunit blockers delay OAT, indicating the involvement of glutamate in OAT. Glycine acts as a co-agonist on NMDARs, and glycine transporters (GlyTs), particularly GlyT-1 inhibitors, could affect the NMDAR pathways related to OAT. Chronic subcutaneous treatments with morphine and NFPS, a GlyT-1 inhibitor, reduced morphine antinociceptive tolerance (MAT) in the rat tail-flick assay, a thermal pain model. In spinal tissues of rats treated with a morphine–NFPS combination, NFPS alone, or vehicle-comparable changes in µ-opioid receptor activation, protein and mRNA expressions were seen. Yet, no changes were observed in GluN2B mRNA levels. An increase was observed in glycine and glutamate contents of cerebrospinal fluids from animals treated with a morphine–NFPS combination and morphine, respectively. Finally, GlyT-1 inhibitors are likely to delay MAT by mechanisms relying on NMDARs functioning rather than an increase in opioid efficacy. This study, to the best of our knowledge, shows for the first time the impact of GlyT-1 inhibitors on MAT. Nevertheless, future studies are required to decipher the exact mechanisms.

## 1. Introduction

Despite the large amount of research and analgesic drugs’ availability, chronic pain has not been satisfactorily solved thus far. Chronic pain, including most cancer pain, is defined by the International Association for the Study of Pain (IASP) as pain that persists or recurs for over three months.

The WHO ladder-based approach for the treatment of cancer pain encompasses nonsteroidal anti-inflammatory drugs (NSAIDs), minor analgesics (paracetamol), and opioid analgesics. NSAIDs can alleviate mild to moderate acute pain. However, in severe acute and some chronic pain conditions (e.g., cancer pain), NSAIDs alone or other minor analgesics are not effective enough in reducing the pain, thus, clinicians combine them with opioid analgesics or prescribe high-efficacy opioid analgesics [1,2].

Opioid analgesics have traditionally been and continue to be among the most effective pain relievers in clinical settings. However, the ongoing opioid epidemic and side effects, including respiratory depression, constipation, and the quick development of analgesic tolerance, have limited their therapeutic utility [3,4,5,6,7]. In chronic pain conditions, progression in the pain intensity along with the development of opioid tolerance can lead to the failure of treatment with opioids, as dose escalation can bring fatal outcomes. In addition, in certain chronic non-cancer pain conditions (neuropathic pain), opioid analgesics’ use is still controversial regarding their efficacy; therefore, opioids have been pushed back to second- and third-line medications, yet represent a substantial risk factor for subsequent opioid use disorders in the younger population [8]. Neuropathic pain is defined by IASP as a pain caused by a lesion or disease of the somatosensory nervous system. The current first-line medications include tricyclic antidepressants, serotonin and norepinephrine reuptake inhibitors, gabapentinoids, and lidocaine transdermal patches [9]. All of these drugs are considered adjuvant medications in the treatment of cancer pain, particularly when the pain is associated with comorbidities, indicating that opioid analgesics are the main pillar in the management of cancer pain. Therefore, there is a need for effective solutions to reduce the side effects of opioid analgesics, including opioid analgesic tolerance (OAT).

Opioids bind to their receptors, namely, μ-opioid receptors (MORs), δ-opioid receptors (DORs), and κ-opioid receptors (KORs) on spinal cord neurons, inhibiting the ascending pain pathway to the brain, thus attenuating pain sensation. Nevertheless, functional opioid receptors located on the descending pain pathway, supraspinal pain regions, and periphery are also implicated in pain modulation [10,11,12,13,14]. In fact, MORs mediate both the opioids’ analgesic effects and their adverse effects, including OAT. The mechanisms underlying the development of OAT have been the subject of several opioid research groups over the past decades, yet the key mechanism remains incompletely understood so far. Nevertheless, underlying mechanisms include desensitization of the MORs and consequent internalization, and, in the long term, downregulation of the receptors [15,16,17,18,19].

N-methyl D-aspartate receptors (NMDARs) are part of the ligand-gated ion channel family as ionotropic glutamate receptors. They are located mostly at excitatory synapses, and thereby participate in excitatory neurotransmission in the central nervous system (CNS). Simultaneous binding of glutamate and co-agonist glycine is required for efficient activation [20,21]. NMDARs are heteromers composed of GluN1, GluN2A, GluN2B, GluN2C, GluN2D, GluN3A, and GluN3B subunits [22]. At the spinal level, NMDARs are localized in the substantia gelatinosa of the dorsal horn with a limited presence elsewhere in the spinal grey matter. Interestingly, they co-localize with MORs within the spinal cord dorsal horn lamina II and brain areas such as the periaqueductal gray, caudate-putamen nucleus, and nucleus accumbens. This co-localization associates MORs and NMDARs in a reciprocal regulation manner [22].

The implication of NMDAR in the development of OAT is traced back to studies that have shown that NMDAR becomes activated in animals treated chronically with morphine, and drugs that inhibit NMDAR delay the development of OAT [23,24]. The co-administration of morphine and various NMDAR antagonists, such as MK801, dextromethorphan, ketamine, and phencyclidine, shows a preventive effect in morphine-tolerance development in animal models [14,25]. Interestingly, MK801 failed to prevent the development of tolerance against fentanyl or DAMGO [26]. Several studies have shed light on an increase in the activity of presynaptic NMDARs of the spinal cord primarily afferent in animals with MAT [27,28,29]. It has also been documented that animals with neuropathic or inflammatory pain, as well as those receiving chronic morphine treatment, exhibit elevated expressions of the GluN2B subunit in supraspinal brain regions and at spinal cord levels [30,31].

Promising outcomes with glycine transporter (GlyT) inhibitors were demonstrated in a rat model of neuropathic pain by our earlier research and other studies [32,33,34]. GlyTs are substantial in the regulation of synaptic and extrasynaptic glycine concentration. The two types, GlyT-1 (expressed on astrocytes and postsynaptic neurons) and GlyT-2 (expressed only on neurons), are found in the brain and spinal cord [35]. Glycine is considered one of the main inhibitory neurotransmitters in the CNS and a co-agonist of NMDA receptors. In neuropathic pain conditions, this inhibitory transmission is hindered in the dorsal horn of the spinal cord, characterized by the hypofunction of glycinergic interneurons located in laminae II and III of the spinal dorsal horn [36]. GlyT-1 is responsible for extrasynaptic glycine concentration regulation by bidirectional transportation and limiting NMDA receptor-mediated excitatory neurotransmission [21,22,35]. Glycinergic neurons are mainly interneurons that modulate the pain sensation pathway. Glycine is released upon action potential in the presynaptic neuron, then diffuses towards the postsynaptic neuron, where it binds to inhibitory glycine receptors. The remaining glycine in the synaptic cleft is taken up by GlyT-2, while GlyT-1 transports glycine back from the extrasynaptic space [21,37]. Our group’s recent study demonstrated that GlyT-1 and Glyt-2 selective inhibitors, NFPS and Org25543, respectively, in a mononeuropathic pain model of rat partial sciatic-nerve ligation (pSNL) produced an acute antiallodynic effect in higher doses (4 mg/kg) [32]. In chronic treatment, a lower dose of NFPS was enough to produce an antiallodynic effect.

In the present work, we provide, to the best of our knowledge, the first evidence for selective GlyT-1 inhibitor NFPS to hinder the development of OAT when co-administered with morphine and also explore the possible underlying molecular mechanisms.

## 2. Results

### 2.1. Behavioral Studies

#### 2.1.1. NFPS Induces Delayed Morphine Antinociceptive Tolerance Development

The antinociceptive effect of morphine, NFPS, and their combination was assessed by the tail-flick thermal pain model on the first day of the experiment, acutely, and chronically after 10 days of subcutaneous (sc.) treatments. Based on our previous study, the treatment schedule and morphine dosage were administered to induce morphine antinociceptive tolerance (MAT) (as described in [38]).

**Figure 1:**
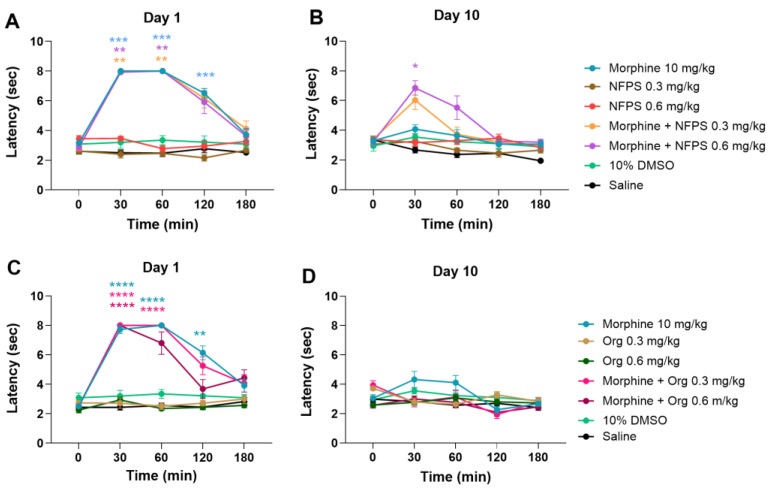
The antinociceptive effect of sc. morphine, NFPS, and Org-25543 and their combination acutely (**A**,**C**) and after 10-day treatment (**B**,**D**). Means ± S.E.M. of tail-flick latency are presented at the tested time points. * *p* < 0.05, ** *p* < 0.01, *** *p* < 0.001, **** *p* < 0.0001 compared to the corresponding vehicle group (morphine vs. saline; NFPS or Org-25543 vs. 10% DMSO; combination vs. saline and 10% DMSO, showing the stricter) in a Mixed-effect model (for panel **D**) and two-way ANOVA (panel, **A**–**C**), followed by Tukey’s multiple comparison test, *n* = 5–12 per group.

Figure 1A,B depicts that acute treatment with sc. 10 mg/kg morphine produces a significant antinociceptive effect; however, after a 10-day treatment, the antinociceptive effect is largely decreased, indicating the development of MAT. On the other hand, NFPS in doses of 0.3 and 0.6 mg/kg, similar to vehicles 10% DMSO and saline, fails to show antinociception either after acute or chronic treatment (Figure 1A,B). Acute co-treatment with NFPS in test doses fails to alter the effect of morphine on the first day of the experiment (Figure 1A). On the other hand, chronic co-treatment with 0.6 mg/kg NFPS and morphine significantly ameliorates the morphine-induced tolerance to antinociception on the 10th day at the peak effect time of morphine (Figure 1B). On the other hand, Org-25543, a GlyT-2 selective inhibitor, was also tested at doses of 0.3 and 0.6 mg/kg to see if it can affect MAT in a manner comparable to the applied NFPS doses. Chronic subcutaneous Org-25543 treatment did not prevent the development of MAT in either of the studied doses (Figure 1C,D). These results suggest that NFPS, but not Org-25543, delays the development of tolerance to the antinociceptive effect of morphine under the present experimental conditions.

#### 2.1.2. Effects of NFPS on Motor Function in the Rat Rotarod Test

**Figure 2:**
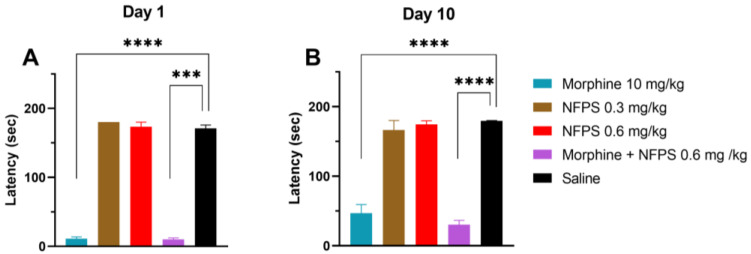
Motor coordination effects of test compounds, morphine, NFPS, and their combination acutely (**A**) and after 10-day treatment (**B**) 30 min after subcutaneous treatment. Columns represent latency times of animals on the rotarod test, shown as mean ± S.E.M., *** *p* < 0.001, **** *p* < 0.0001 compared to saline; Kruskal–Wallis test with uncorrected Dunn’s post hoc test, *n* = 5–17 per group. Day 1: H = 45.06; Day 10: H = 40.59.

Chronic treatment with either 0.3 mg/kg or 0.6 mg/kg NFPS does not change the rats’ motor function 30 min after subcutaneous administration (the peak effect time of morphine), whereas morphine per se or in combination with 0.6 mg/kg NFPS induces significant motor dysfunction (Figure 2A). After 10 days, as morphine tolerance developed, the decreased motor coordination in the morphine group was partially regained and remained unchanged when combined with 0.6 mg/kg NFPS (Figure 2B).

### 2.2. In Vitro Studies

#### 2.2.1. The Effect of NFPS on the Level of Glycine and Glutamate in the Cerebrospinal Fluid in Rats Treated Chronically with Morphine

**Figure 3:**
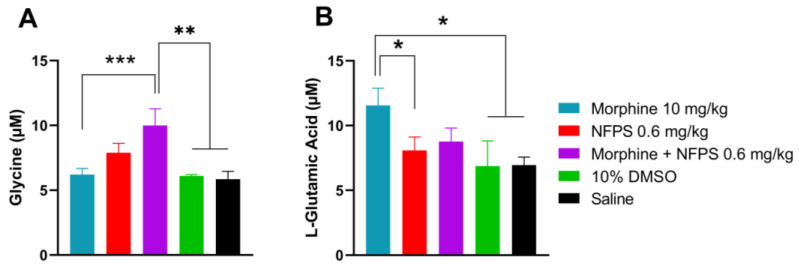
CSF glycine (**A**) and glutamate (**B**) content of animals after 10-day subcutaneous treatments with morphine, NFPS, or their combination. Data are presented as means ± S.E.M. in µM concentration. * *p* < 0.05, ** *p* < 0.01, *** *p* < 0.001 vs. vehicles, one-way ANOVA followed by Fisher’s LSD test, *n* = 6–22 per group. ((F (4, 28) = 5.116 for panel **A**; F(4, 53) = 2.610 for panel **B**).

Our previous studies indicated the crucial role of glycine and glutamate in the development of altered pain sensation as a consequence of the imbalance between the excitatory and inhibitory system at the spinal levels [34,39]. Thus, these experiments were intended to determine the content of glycine and glutamate in the CSF by the capillary electrophoresis method developed in our laboratory [40]. CSF samples were taken from animals after the 10-day treatment. The CSF glycine content showed an increase in all NFPS-treated animals and was significant in the morphine- and 0.6 mg/kg NFPS-treated group compared to both vehicles (Figure 3A). The glutamate level was also significantly increased in the morphine-treated group compared to the group treated with vehicles or NFPS per se (Figure 3B). On the other hand, animals treated with the morphine–NFPS combination showed a glutamate level comparable to that measured for vehicle-treated groups (DMSO and saline) (Figure 3B).

#### 2.2.2. The Effect of NFPS on Morphine Efficacy in Spinal Tissues in Rats Treated Chronically with Morphine

**Figure 4:**
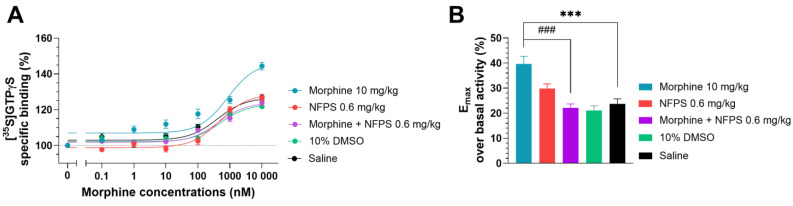
The effect of chronic treatment with NFPS (0.6 mg/kg, sc.) alone or in combination with morphine (10 mg/kg sc.) on MOR agonist-induced G-protein activity in morphine-induced [^35^S]GTPγS binding in homogenized L4-6 sections of spinal cord from corresponding treated animals (**A**). Concentration–response curves of morphine-induced [^35^S]GTPγS binding measured in samples from the indicated treatment groups. Figure 4A represents the specific binding of [^35^S]GTPγS in the presence of increasing concentrations (0.1 nM–10 μM) of morphine. Points represent means ± S.E.M. for at least three experiments performed in triplicate. “Basal” on the x-axis indicates the basal activity of the monitored G-protein, which is measured in the absence of morphine and also represents the total specific binding of [^35^S]GTPγS. The level of basal activity was defined as 100%, indicated by dotted line (**B**). Maximum efficacy of morphine over basal activity calculated from concentration–response curves. Columns represent means ± S.E.M. ***/###: *p* < 0.001, one-way ANOVA with Dunnett’s multiple comparisons test (compared to saline-treated group (F (4, 50) = 12.16); compared to morphine 10 mg/kg alone-treated group (F (6, 77) = 10.66)).

In this section, a G-protein activity-binding test was used to measure the morphine’s ability to activate MORs in the spinal tissues of groups that had received chronic treatments. In the saline-treated group, morphine displayed 23.74% ± 1.97 (*n* = 10) maximum efficacy (E_max_) over basal activity (Figure 4B), which corresponds well with our previous measurements obtained in a similar setup [41]. Chronic DMSO treatment did not alter the efficacy of morphine (21.09% ± 1.85, *n* = 10; one-way ANOVA with Dunnett’s multiple comparisons test). The E_max_ value of morphine in the spinal tissues of rats treated chronically with NFPS per se (0.6 mg/kg, Figure 4B) did not differ significantly from that obtained in the group that received saline treatment (29.85 % ± 1.83, *n* = 10; one-way ANOVA with Dunnett’s multiple comparisons test). The E_max_ value of morphine in spinal cord samples from animals receiving chronic morphine treatment (10 days, 10 mg/kg sc.) was significantly higher compared to the saline-treated group (39.67 % ± 3.06, *n* = 15, Figure 4B, ***: *p* < 0.001, one-way ANOVA with Dunnett’s multiple comparisons test).

The elevated efficacy (E_max_) of morphine in spinal tissues from animals treated chronically with morphine per se was restored in spinal cord samples from animals receiving simultaneous and chronic treatment with morphine and 0.6 mg/kg NFPS combination (22.14% ± 1.56, *n* = 10, ***: *p* < 0.001, respectively, one-way ANOVA with Dunnett’s multiple comparisons test).

#### 2.2.3. The Effect of NFPS on µ-Opioid Receptor Protein Level in Spinal Tissues in Rats Treated Chronically with Morphine

**Figure 5:**
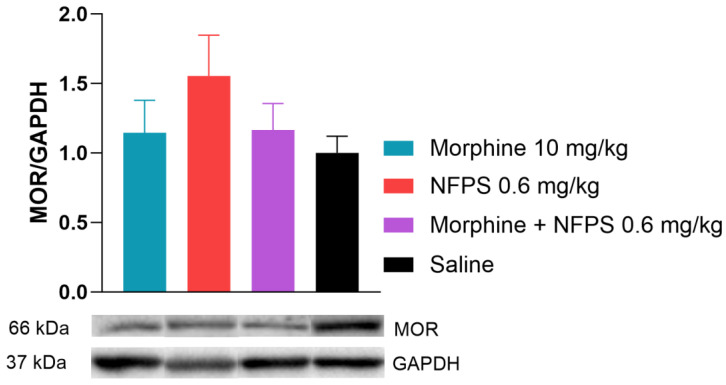
Spinal cord MOR levels relative to GAPDH after 10-day treatment. Representative bands are taken from the same membrane. Data are presented as means ± S.E.M. *p* > 0.05 vs. saline, one-way ANOVA followed by Dunnett’s post hoc test, *n* = 9–10 per group.

In this section of the investigation, rats receiving chronic treatments with morphine, NFPS, a morphine–NFPS combination, and saline had their spinal tissues examined using Western blotting to determine if there is an alteration in the protein level of MOR in response to chronic morphine treatment. We found that the MOR protein expression did not change after chronic morphine treatment alone or in combination with NFPS (Figure 5). It is worth noting that NFPS per se also showed an increase in the MOR expression, but this alteration was not significant (Figure 5).

#### 2.2.4. The Effect of NFPS on µ-Opioid Receptor and GluN2B mRNA Expression in Spinal Tissues in Rats Treated Chronically with Morphine

**Figure 6:**
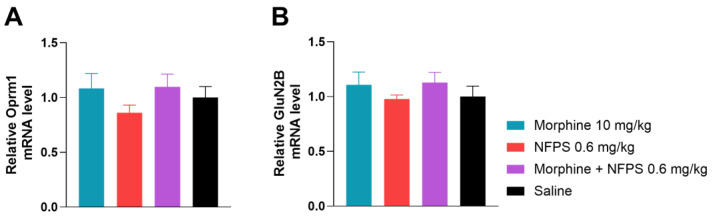
The effect of 10-day morphine, NFPS and combination treatment on spinal cord Oprm1 and GluN2B expression relative to GAPDH. Data is presented as means ± S.E.M. For statistical analysis One-way ANOVA followed by Dunnett’s post hoc test was used, *n* = 5 per group.

To gain a better understanding of what changes take place after the 10-day treatment with morphine, NFPS, and their combination on the gene regulatory level, we first investigated Oprm1 mRNA levels (Figure 6A), applying qRT-PCR analysis. In this regard, we found no significant changes between the test groups.

Finally, we assessed GluN2B mRNA levels of the same animals and found no significant difference between treatment groups (Figure 6B). This led us to the conclusion that the chosen 10-day treatment period does not alter gene expression of either Oprm1 or GluN2B.

## 3. Discussion

The effect of NFPS, a GlyT-1 inhibitor, on the development of MAT in the rat tail-flick assay, a thermal pain model, is the focus of our investigation. To the best of our knowledge, this is the first time this effect has been shown. NFPS is a selective inhibitor of GlyT-1 with a long-lasting action [42,43]. The current widely accepted consensus is that GlyT-1 is located on astrocytes, where it functions to clear the extrasynaptic glycine, though its neuronal distribution has also been reported [37,44]. As mentioned in the introduction, glycine is also an important co-agonist for NMDARs, namely GluN2A, GluN2B, GluN2C, and GluN2D [22]. It has also been observed that glycine is the preferred co-agonist by NMDRs containing GluN2B subunits [21]. In fact, glycine is an amino acid that displays both inhibitory and excitatory functions; the former is a consequence of glycine receptor activation, whereas the latter is a consequence of its co-agonist effect on NMDA receptors’ function [34,45]. Notably, GlyT-1 can change the concentration of glycine in the vicinity of GluN2B by acting in either a forward or reverse manner [22,45,46]. Several studies have suggested the substantial role of astrocytic GlyT-1 in the regulation of extrasynaptic glycine levels; thus, upon its inhibition, the increase in glycine concentrations predominantly occurs in the extrasynaptic region [35,45,46]. On the other hand, a large amount of evidence has shed light on the fact that spinal GlyT-2 expression is restricted to the glycinergic synapses and is functioning to regulate the concentration of glycine present in synapses as well as the refilling of presynaptic vesicles. In fact, among the synaptic glycinergic receptors, the α3β GlyRs have been demonstrated to mediate pain-inhibitory effects. They are found in laminae I and II of the spinal dorsal horn, a major pain relay point [37,47]. Strychnine-sensitive glycine receptors are implicated in the disinhibitory process, which occurs in the dorsal horn of the spinal cord following damage to peripheral primary sensory neurons. It is interesting to note, as an example, that the disinhibitory process is principally mediated by the spinal GABArgic system, stemming from both a reduction in the content of GABA and its receptors. In addition, the colocalization of GABA and synaptic glycine receptors is previously described. However, to the best of our knowledge, there is no clinical evidence to support the use of GABA-potentiating medications to slow the onset of opioid analgesic tolerance. Rather, issues with side effects have been brought up regarding the co-administration of GABA-mimicking medications and opioid analgesics. Nevertheless, dysfunction in spinal cord glycinergic and GABAergic systems are involved in the development of allodynia and hyperalgesia associated with neuropathic and inflammatory pains [48]. GlyT-2 inhibitors do, in fact, have an antinociceptive effect, as previous studies have demonstrated, but the antinociceptive dose also causes notable adverse effects, such as respiratory depression and motor impairments, indicating the narrow therapeutic window [49]. To discover whether the GlyT-2 inhibitor delays the development of OAT, we have also tested the impact of Org-25543, a GlyT-2 inhibitor, in doses proven to have an analgesic effect in animal models [50]. Org-25543 failed to delay MAT in the applied doses, which were in the safe dose range in terms of side effects [32]. On the other hand, when morphine and NFPS, a GlyT-1 inhibitor, were administered to rats simultaneously, MAT development was delayed. This result is close to our hypothesis regarding the effect of the extrasynaptic glycine level on OAT in the current investigation. As a result, we have limited our attention in the current investigation to the impact of GlyT-1 inhibitors in MAT.

Based on behavioral results, our present study also examines the possible mechanisms that may be involved in the delaying of MAT in rats upon systemic chronic simultaneous administration of NFPS and morphine. The questions posed were whether GlyT-1 inhibitors contribute to the preservation of MOR sensitivity to morphine and if glycine levels are affected by such inhibitor treatments in the current experimental setup. Though merely a tendency toward glycine elevation was observed after NFPS treatment, the answer to the later issue was supported by the animals’ elevated glycine levels after NFPS or the NFPS–morphine combination treatment. Spinal samples from every group, with the exception of the morphine-treated group, demonstrated comparable morphine sensitivity in the G-protein activation assay. As evidenced by Emax values, morphine displayed significant efficacy in the spinal sample from the morphine-treated group (Figure 4). These findings corroborate earlier research using the same assay ([35S]GTPγS binding), which showed that morphine-induced tolerance was not linked to a decrease in G-protein activation in spinal samples from mice, who were tolerant to morphine [51]. Additionally, this work has demonstrated a variation in G-protein activation following the administration of several MOR agonists. In this regard, the efficacy of fentanyl was decreased, whereas that of morphine was increased. Numerous signaling effectors triggered by MOR stimulation have been identified by other studies, among them, extracellular signal-regulated kinase (ERK), which has been linked to the activation of NMDARs and glutamate release [52,53,54,55]. From these results, we had supposed that the impact of GlyT-1 inhibitors on the delaying of MAT was largely dependent on their effect on NMDAR function.

The molecular aspect of NFPS-induced GlyT-1 inhibition reveals the increase in the glycine level in CSF. To determine whether this elevation reflects the conditions in the vicinity of NMDARs, future studies are needed. Drugs that inhibit NMDARs in a competitive or noncompetitive manner, as well as glycine binding-site antagonists or partial agonists, have been demonstrated to delay the onset of MAT [56,57,58,59,60,61]. In addition, several studies have pointed to the crucial role of NMDAR-activation in OAT development at pain-processing key points, including the spinal dorsal horn [62,63,64]. Opioid-induced MORs’ activation stimulates NMDARs through protein kinase C (PKC) and Src by phosphorylating the receptor at the C-termini of GluN2A and GluN2B subunits, which then increases the calcium permeability of the neuron. In addition, such enhanced calcium levels increase the activity of Ca^2+^/calmodulin-dependent protein kinase II (CaMKII) and nitric oxide synthase (NOS), where CaMKII desensitizes MORs via receptor phosphorylation, while NOS stimulates nitric oxide production, which eventually may increase glutamate release [65]. Such a mechanism generates a cycle of sustained NMDAR activation and MOR desensitization, which contributes to the development of OAT. GluN2B has been implicated in OAT in the spinal dorsal horn, and selective GluN2B inhibitors (antagonists or negative allosteric modulators) are promising candidates for reducing OAT [66]. The increase in glutamate release has also been postulated in the animals that underwent chronic morphine treatment [62]. This was the case in our study, where we found a significant enhancement of glutamate levels in the CSF of animals treated chronically with morphine (Figure 3B). Considering the distribution of NMDARs, specifically GluN2B, which has been reported to be located in the extrasynaptic region and preferring glycine as a co-agonist, we would expect an alteration in GluN2B function [21,22,35]. Furthermore, studies conducted by other groups have shown the synaptic and extrasynaptic distribution of NMDAR containing a GluN2B subunit [55,67]. Ivanov and colleagues have demonstrated that extrasynaptic GluN2B activation is implicated in ERK dephosphorylation and inactivation, whereas synaptic GluN2B phosphorylates and activates ERK [55]. It means that in physiological conditions, there is a balance between the effects of extrasynaptic and synaptic GluN2B. However, during pathological conditions or drug-induced ERK activation, as in the case of sustained treatment with morphine, its activation might be implicated in the development of OAT [52,55]. It is important to note that an increase in the extrasynaptic concentration of glycine or D-serine, the main co-agonists of these receptors, is the prerequisite for more extrasynaptic GluN2B activation.

The administration of NFPS results in an increase in the glycine level in the CSF once administered per se or combined with morphine. Given the fact that the operation region gives a reason to suspect that the blockade of GlyT-1 results in an increase in glycine level in the vicinity of GluN2B alongside glutamate, this can direct the receptor to either overactivity or the internalization process. The activation of GluN2B under the present scenario might result in ERK inactivation. However, previous studies have also shown the significance of glycine in relation to its function in ion channel opening and likely in the internalization of the receptor from the cell surface [20,22,68,69]. In fact, in our present study, it is unclear whether internalized NMDAR receptors are from the extrasynaptic, synaptic, or both regions. In this regard, Nong et al. have pointed to the internalization of both synaptic and extrasynaptic NMDARs when both glutamate and glycine levels are increased [68]. In addition, it is important to recognize the intricacy of glutamatergic neuronal connections within the CNS that can mediate simultaneous activation and inhibition.

Initially, we anticipated a deleterious effect on pain perception that comes next to receptor activation, a process that occurs prior to the receptor internalization being described for GluN2B. Indeed, the perception of pain in animals could not justify overactivation. The CSFs of rats exhibiting delayed MAT showed a significant increase in the levels of both glutamate and glycine, which denies the consequences of extrasynaptic GluN2B activation previously hypothesized [22]. It is possible that the observed effects of the morphine and NFPS combination on OAT are due to either ERK inactivation or the internalization of extrasynaptic GluN2B. In this context, we have previously demonstrated that a rise in glycine concentrations may decrease the NMDA current, and investigations conducted by other groups have demonstrated the internalization or downregulation of receptors in similar circumstances [22,68,69,70,71]. A previous study by Bhargava and colleagues has shown that in the spinal cord of rats tolerant to morphine, a simultaneous increase in glutamate and glycine levels leads to the downregulation of NMDARs [71]. Moreover, the abovementioned data on the discrepancies between signal pathways that play a role in the activation of ERK, an important cellular effector that activates next to MOR activation, have been reported. In this regard, ERK is triggered by GPCR following morphine treatment [72]. On the other hand, etorphine is likely to trigger ERK through the β-arrestin-dependent pathway. ERK activation is involved in the release of glutamate under tolerance conditions. As mentioned above, stimulation of the extrasynaptic NMDARs containing the GluN2B subunit has been reported to elicit ERK inactivation rather than ERK activation, yet synaptic NMDAR containing GluN2B activates ERK [55]. The efficacy of GlyT-1 inhibitors in delaying the development of MAT may have been operated by this mechanism, as opposed to that of GlyT-2 inhibitors. On the other hand, GlyT-2 inhibitors failed to delay MAT under our present experimental set (Figure 1D), further confirming that the extrasynaptic NMDARs are the target mediating the effect of the GlyT-1 inhibitor. It is evident from all these explanations just how challenging it is to comprehend the current processes by which morphine and GlyT-1 inhibitors work well together to delay the MAT.

To decipher the mechanism, we have also analyzed whether GluN2B mRNA levels have changed under this experimental setup. The results of this set of tests show that no discernible changes could be measured. Previous studies by Gong and colleagues have demonstrated an elevation of NMDAR-containing GluN2B subunits’ protein expression in the dorsal root ganglions (DRGs) of rats that received long-term morphine treatment [25]. These results point to changes in the periphery rather than the CNS, namely the spinal cord. As a matter of fact, the DRGs host the machinery responsible for synthesizing the receptors. Western blot and immunohistochemical assays have been used in another study that is more closely similar to ours, either in terms of animal phenotype or morphine administration, to assess the alterations in the spinal GluN2B [59]. The results of this study have shown that rats chronically treated with morphine have higher levels of GluN2B protein expression and immunoreactivity in their spinal cords. Additionally, the immunohistochemistry findings confirmed that neuronal GluN2B was upregulated, but not that of microglia or astrocytes. Several studies have investigated the involvement of GluN2B subunits containing NMDARs in the development of OAT. Previous behavioral studies have shown that drugs inhibiting GluN2B can delay the development of morphine analgesic tolerance. However, data related to the changes of GluN2B are contradictory, supporting the upregulation, no change, or even downregulation [31,71,73,74,75,76,77].

Furthermore, there were no discernible changes in MOR protein and Oprm1 mRNA levels in relation to the treatments, which is supported by other studies as well [78,79,80,81]. It is worth noting that despite the development of MAT, morphine does not cause MOR downregulation compared to certain opioid agonists like fentanyl [7]. For this reason, it is not possible to infer a connection between the GlyT-1 inhibitors and the spinal MORs in the current study. It remains possible that NFPS drives the delay in MAT via a mechanism that includes GlyT-1 reverse mode inhibition or that it acts as a partial agonist on the glycine binding site of GluN2B, which ultimately creates a balance between pro-nociceptive and antinociceptive pathways. Ultimately, in the context of GluN2B activation, the increased CSF glutamate and glycine concentrations in this study initially appeared contradictory. However, the distribution pattern and opposing effect of this receptor subtype on ERK function, which has a crucial role in the development of MAT, partially revealed the advantageous relationship between extrasynaptic glycine and glutamate with regard to ERK inhibition. Future research, in particular that focuses on the ERK inhibition and NMDAR-containing GluN2B internalization under sustained simultaneous administration of various GlyTs inhibitors and morphine or other opioid agonists, is needed to completely unravel the mechanisms. Limitations: lack of study on the pharmacokinetic interaction between NFPS and morphine is one of the limitations of the present work; thus, future studies are required to justify this issue.

## 4. Materials and Methods

### 4.1. Animals

For in vivo tests and the following receptor binding assays and capillary electrophoresis tests, male Wistar rats (180–250 g body weight) were used. Rats were purchased from Toxi-Coop Zrt. (Budapest, Hungary) and housed in the local animal house of the Department of Pharmacology and Pharmacotherapy, Semmelweis University (Budapest, Hungary).

The animals were kept at a constant temperature of 20 ± 2 °C under a 12:12 light and dark cycle in standard cages, holding four to five animals per cage, and were provided with water and food ad libitum. All housing and experiments were handled in accordance with the European Communities Council Directives (2010/63/EU), the Hungarian Act for the Protection of Animals in Research (XXVIII.tv. 32.§), and a local animal care committee (PEI/001/276-4/2013). All efforts were made to minimize the number of animals and their suffering.

### 4.2. Materials

NFPS and Org-25543 were obtained from Tocris Bioscience via Bio-Techne R&D Systems Kft. (Budapest, Hungary), while morphine-HCl was obtained from Alkaloida-ICN (Tiszavasvári, Hungary). NFPS was dissolved in 10% Dimethyl sulfoxide (DMSO) in 0.9% saline and morphine-HCl was dissolved in 0.9% saline. All compounds were administered subcutaneously (sc.) in a total volume of 2.5 mL/kg bodyweight for morphine and 1.25 mL/kg bodyweight for NFPS and Org25543. NFPS and Org25543 were administered once daily (morning), while morphine was administered twice daily (morning and evening). Diethyl-ether was purchased from Sigma-Aldrich (Budapest, Hungary).

For [^35^S]GTPγS binding assay DMSO, Tris-HCl, EGTA, NaCl, MgCl_2_ × 6H_2_O, GDP, and the GTP analog GTPγS were purchased from Sigma-Aldrich (Budapest, Hungary). The radiolabeled GTP analog, [^35^S]GTPγS (specific activity: 1000 Ci/mmol), was obtained from Hartmann Analytic via Izotóp Intézet Kft (Budapest, Hungary). The UltimaGoldTM MV aqueous scintillation cocktail was purchased from PerkinElmer (handled by Per-Form Hungaria Kft, Budapest, Hungary). All compounds were stored and handled as described in the product information sheets.

### 4.3. Thermal Acute Pain Model (Rat Tail-Flick) and the Induction of Morphine Antinociceptive Tolerance

The rat tail-flick test, a thermal pain model, is widely used to assess both the analgesic effect of opioid agonists as well as the development of OAT. In order to create MAT, the experimental setup and morphine treatment protocol were followed according to earlier studies [23,38]. Therefore, this assay was performed in order to analyze the antinociceptive effect of NFPS, Org25543, morphine, and their combination in a rat tolerance model. Briefly, a light beam (IITC Life Science, Woodland Hills, CA, USA) is focused on dorsum of the lower third of the rat tail, and the latency time (s) to flick its tail is measured as a pain threshold. In the days preceding the start of the experiments, handling was performed to acclimatize the animals to the experimental conditions. For these experiments, rats were treated with sc. morphine (10 mg/kg), NFPS, Org25543 (0.3 and 0.6 mg/kg), or with their combination (10 mg/kg + 0.3 mg/kg or 0.6 mg/kg). As a control, 0.9% saline or 10% DMSO was used. The pain threshold of animals was determined prior to and 30, 60, 120, 180 min after treatment on days 1 and 10. Eight seconds was used as a cut-off time in order to avoid tissue damage. After the last measurement, animals were sacrificed by diethyl-ether overdose, and cerebrospinal fluid (CSF) and spinal cord samples were obtained for further in vitro analyses.

### 4.4. Rotarod Test

The possible motor function side effects were assessed via the rotarod test (Rat RotaRod, model 7750, Ugo Basile, Italy) on the 1st and 10th day, as described previously [39]. On the day preceding the experiment, animals were trained to stay on the rotating rod for 180 s. Rotation speed was set at 16 rotations per minute (RPM). Animals’ motor function was assessed 30 min following treatment with 10 mg/kg morphine, 0.3 mg/kg and 0.6 mg/kg NFPS, morphine, and 0.6 mg/kg NFPS combination and 0.9% saline. The cut-off time was 180 s, otherwise the fall-off (latency) time was noted.

### 4.5. Capillary Electrophoresis

After 10-day treatment, animals’ CSF was collected, and glycine and glutamate levels were measured by capillary electrophoresis as described previously [39,40]. CSF samples were obtained by cisterna magna puncture and then centrifuged at 2000× *g* at 4 °C for 10 min. Samples were placed at −80 °C immediately and stored there until further processing. On the day of measurement, samples were deproteinized by mixing with 2 volumes of pure acetonitrile and centrifuged at 20,000× *g* for 10 min at 4 °C. Supernatants were collected and diluted five times with acetonitrile–distilled water solution (2:1; *v*/*v*). Samples were subjected to derivatization with 7-fluoro-4-nitro-2,1,3-benzoxadiazole (NBD-F, Tokyo Chemical Industries, Tokyo, Japan) at 1 mg/mL final concentration in 20 mM borate buffer pH 8.5 for 20 min at 65 °C. Five µM L-cysteic acid was used as internal standard. Derivatized samples were analyzed using a P/ACE MDQ Plus capillary electrophoresis system coupled with a laser-induced fluorescence detector (Sciex, Framingham, MA, USA). Excitation and emission wavelengths were 488 and 520 nm, respectively. Separation was carried out using polyacrylamide-coated fused-silica capillaries (i.d.: 75 µm, effective/total length: 50/60 cm) with 50 mM HEPES running buffer pH 7.0 containing 6 mM hydroxypropyl amino-β-cyclodextrin at 15 °C by applying −27 kV constant voltage.

### 4.6. G-Protein Receptor Binding Assay

#### 4.6.1. Spinal Cord Sample Preparations

Animals were decapitated and their spinal cords of L4-6 sections were quickly removed. The tissue samples were prepared for membrane preparation according to our previous work [41]. In brief, samples were homogenized in ice-cold TEM (Tris-HCl, EGTA, MgCl_2_) (pH 7.4) buffer and stored at −80 °C for further use.

#### 4.6.2. Functional [^35^S]GTPγS Binding Assays

In [^35^S]GTPγS binding experiments, we measure the GDP→GTP exchange of the Gαi/o protein in the presence of the test compound in increasing concentrations to measure ligand potency and the maximal effect (efficacy) of receptors’ G-protein. The nucleotide exchange is monitored by a radioactive, non-hydrolysable GTP analog, [^35^S]GTPγS.

The functional [^35^S]GTPγS binding experiments were performed as previously described, with modifications. Briefly, the rat spinal cord membrane homogenates containing ~10 μg/mL protein were incubated at 30 °C for 60 min in Tris-EGTA buffer (pH 7.4) composed of 50 mM Tris-HCl, 1 mM EGTA, 3 mM MgCl_2_, and 100 mM NaCl. The incubation mixture also contained 0.05 nM [^35^S]GTPγS, 30 µM GDP, and increasing concentrations (0.1–10 µM) of morphine.

Total binding was measured in the absence of the agonist, while non-specific binding was determined in the presence of 10 µM unlabeled GTPγS. The bound and unbound [^35^S]GTPγS were separated through Whatman GF/B glass fibers (GE Healthcare Life Sciences through Izinta Kft. Budapest, Hungary) by rapid vacuum filtration and by washing the samples with ice-cold 50 mM Tris-HCl buffer (pH 7.4). The radioactivity of the filters was detected by UltimaGoldTM MC aqueous scintillation cocktail with Packard Tricarb 2300 TR liquid scintillation counter. [^35^S]GTPγS binding experiments were performed in triplicates and repeated at least three times.

### 4.7. Western Blot Analysis

Spinal cord tissues were homogenized with a TissueLyser (Qiagen, Venlo, The Netherlands) in lysis buffer supplemented with a protease inhibitor cocktail (cOmplete ULTRA Tablets, Roche, Basel, Switzerland) and PMSF (Sigma, St. Louis, MO, USA). The homogenates were centrifuged twice at 1500× *g* and 4 °C for 15 min, the supernatants were collected, and their protein concentration was measured by the bicinchoninic acid assay (BCA, Thermo Fisher Scientific, Waltham, MA, USA). An equal amount of protein (35 µg) was mixed with Pierce Lane Marker reducing sample buffer (Thermo Fisher Scientific, Waltham, MA, USA) and loaded and separated in a 4–20% precast Tris-glycine SDS polyacrylamide gel (BioRad, Hercules, CA, USA). Proteins were transferred electrophoretically onto a polyvinylidene difluoride membrane (BioRad, Hercules, CA, USA) at 200 mA overnight. Membranes were blocked with 5% nonfat dry milk (BioRad, Hercules, CA, USA) in Tris-buffered saline containing 0.05% Tween-20 (0.05% TBS-T; Sigma, St. Louis, MO, USA) at room temperature for 1 h. Membranes were incubated with primary antibodies against MOR (ABIN617908, 1:1500) (antibodies-online GmbH, Aachen, Germany) overnight at 4 °C, followed by 2 h incubation at room temperature with an appropriate secondary antibody. GAPDH (D16H11, 1:10,000, Cell Signaling Technology, Danvers, MA, USA) was used to control for sample loading and protein transfer and to normalize the content of target protein. At least three repetitions were performed for each experiment. Signals were detected with a chemiluminescence kit (BioRad, Hercules, CA, USA) by Chemidoc XRS+ (BioRad, Hercules, CA, USA) [82].

### 4.8. qRT-PCR

Total RNA was obtained from 10–30 mg of spinal cord tissue using the QIAzol extraction method (Qiagen, Hilden, Germany). RNA concentration was measured with a Nanophotometer (Implen GmbH, Munich, Germany). Reverse transcription was performed from 1 μg of total RNA with a Sensifast cDNA synthesis kit (Bioline, London, UK) according to the manufacturer’s protocol. Target genes were amplified using a LightCycler^®^ 480 II instrument (Roche, Germany) using the SensiFAST SYBR Green master mix (Bioline, UK). Expression levels were calculated with the 2^–ΔΔCT^ evaluation method, and GAPDH was used as reference gene. The sequences of primers used for determination are as follows: MOP (for: GACCGTTTCCTGGCACTTCT; rev: TAGGGCAATGGAGCAGTTTCT), GluN2B (for: AGCTGTTTGGAGATGGGGAG; rev: CTTGCCAGAACAGACACCCA), GAPDH (for: TACCAGGGCTGCCTTCTCTTG; rev: GGATCTCGCTCCTGGAAGATG) [82].

### 4.9. Statistical Analysis

All values are presented as mean ± standard error of means (S.E.M.). Statistical analysis was performed with the GraphPad Prism software (version 8.0.1; GraphPad Software Inc., San Diego, CA, USA). To determine antinociceptive effect and statistical significance, tail-flick data was tested with a Mixed-effect model or two-way ANOVA followed by Tukey’s multiple comparison test. In the case of CSF glycine levels, one-way ANOVA followed by Fisher’s LSD multiple comparison test was used. For the rotarod tests, the Kruskal–Wallis test followed by Dunn’s post hoc test was used. In case of receptor binding data, one-way ANOVA followed by Dunnett’s post hoc test was utilized. For the Western blot and qRT-PCR analysis, one-way ANOVA was applied. Statistical significance was accepted at *p* < 0.05. ROUT analysis was performed to identify outliers, with *Q* value = 0.5%.

## Data Availability

The data presented in this study are available on request from the corresponding authors.
